# Identification of Novel Bioactive Molecules in Black Chiloe’s Giant Garlic (*Allium ampeloprasum* L.) by Green Microwave-Assisted Extraction and Effect-Directed Analysis Using High-Performance Thin Layer Chromatography-Bioassay and Mass Spectrometry

**DOI:** 10.3390/antiox14080913

**Published:** 2025-07-25

**Authors:** Joaquín Fernández-Martínez, David Arráez-Román, Darlene Peterssen, Gerald Zapata, Karem Henríquez-Aedo, Mario Aranda

**Affiliations:** 1 Laboratory of Food and Drug Research, Faculty of Chemistry and Pharmacy, Pontificia Universidad Católica de Chile, Santiago 7820436, Chile; jefernandez2@uc.cl; 2 Department of Analytical Chemistry, Universidad de Granada, 18071 Granada, Spain; darraez@ugr.es; 3 Department of Biological and Chemical Sciences, Faculty of Sciences, Universidad San Sebastián, Concepción 4090762, Chile; darlene.peterssen@uss.cl; 4 Center of Molecular Modelling, Biophysics and Bioinformatics, Faculty of Chemistry and Pharmaceutical Sciences, Universidad de Chile, Santiago 8380544, Chile; gzapata@uchile.cl; 5 Laboratory of Food Biotechnology and Genetics, Department of Basic Sciences, Faculty of Sciences, Universidad del Bío-Bío, Chillán 3810189, Chile

**Keywords:** MAE, (poly)phenols, planar chromatography, S-allyl-cysteine, cyclooxygenase, α-glucosidase, and acetylcholinesterase

## Abstract

Black Chiloe’s giant garlic is a functional food produced by a mild Maillard reaction that contains relevant bioactive molecules like organosulfur compounds (OSCs) and (poly)phenols (PPs). Compared with raw garlic, black garlic has a higher content of PPs and S-allyl cysteine (SAC), a key OSC due to its bioactivities. The objective of the present work was to optimize by chemometric tools a green microwave-assisted extraction (MAE) of SAC and PPs present in black Chiloe’s giant garlic to detect and identify novel bioactive molecules with antioxidant and/or inhibitory activities over cyclooxygenase, α-glucosidase, and acetylcholinesterase enzymes. The MAE factors were optimized using a central composite design, establishing optimal PP and SAC yields at 67 °C, 0% ethanol, 12 min and 30 °C, 40% ethanol, 3 min, respectively. PP and SAC values were 9.19 ± 0.18 mg GAE/g DW and 2.55 ± 0.10 mg SAC/g DW. Applying effect-directed analysis using high-performance thin layer chromatography-bioassay and mass spectrometry, the bioactive molecules present in the MAE extract with antioxidant and inhibitory activities over cyclooxygenase, α-glucosidase, and acetylcholinesterase enzymes were identified as N-fructosyl-glutamyl-S-(1-propenyl)cysteine, N-fructosyl-glutamylphenylalanine, and Harmane.

## 1. Introduction

Chronic non-communicable diseases (CNCDs) are responsible for 74% of all deaths worldwide [1]. The most relevant ones are cancer, type 2 diabetes, and cardiovascular and chronic respiratory diseases, accounting for approximately 80% of all CNCD deaths [1]. Unhealthy diet is one of the most important risk factors for CNCD [1]. This association has driven the study of the relationship between human health and functional foods, finding a key role for this type of foods in lowering the risk of suffering CNCD [2]. Numerous plants, foods, and microorganisms have been studied to identify the presence of different types of bioactive molecules with therapeutical potential, especially those with inhibitory activity over enzymes like α-glucosidase (AG), acetylcholinesterase (AChE), and cyclooxygenase (COX) related to CNCD, such as type 2 diabetes, Alzheimer’s diseases, and cardiovascular pathologies [3]. A well-known functional food is garlic (*Allium sativum*), which is a common spice with several health benefits, including antifungal, antibacterial, antiviral, antioxidant, anti-inflammatory, anticancer, antidiabetic, antihypertensive, and neuroprotective effects [4]. These bioactivities are associated with the presence of organosulfur compounds (OSCs) and (poly)phenols (PPs). The most representative OSCs are S-allyl cysteine (SAC), S-allyl cysteine sulfoxide (alliin), and *γ*-glutamyl-S-allyl cysteine (GSAC), which are responsible for organoleptic and pharmacological properties [5,6]. In the case of PPs, it is possible to find phenolic acids and flavonoids, both of which contribute to antioxidant activity [7] preventing oxidative damage on a cellular level related to CNCD [5,8]. OSCs and PPs are also present in Chiloe’s giant garlic (*Allium ampeloprasum* L.) [5,9], which resembles common garlic in shape, flavor, and bioactive molecules profile but with a bigger bulb size, and different quantities of bioactive molecules [5,10]. Chiloe’s giant garlic is sometimes called great-headed or elephant garlic and locally “*ajo chilote*” because it is micro-cultivated in Chiloé Island (Chile) [11]. This location has been recognized by the Food and Agriculture Organization of the United Nations as a Globally Important Agricultural Heritage Site of the world [12]. Giant garlic has a milder flavor and sweeter taste than common garlic due to the decreased concentration of alliin, which is transformed into allicin, responsible for garlic’s characteristic pungent flavor [6]. Compared with common garlic, giant garlic has a higher concentration of PPs and *γ*-glutamyl peptides, and a lower concentration of volatile OSCs derived from alliin [5,13,14]. One of the compounds derived from *γ*-glutamyl peptides is SAC, which is produced from the hydrolysis of GSAC by the enzyme *γ*-glutamyl transferase (GGT) [15]. SAC is a stable OSC with a similar structure to cysteine showing high-water solubility and low toxicity, both of which are highly desirable properties for the development of functional ingredients and/or nutraceutical products. SAC possesses relevant biological activities, including antioxidant, antidiabetic, neurotrophic, hepatoprotective, and anticarcinogenic effects [16]. This bioactive molecule is present at higher concentrations in aged and black garlics [17,18]. The latter, including black Chiloe’s giant garlic, is a unique product obtained by subjecting whole garlic bulbs to mild Maillard reaction conditions (high temperatures and humidity) over a period of 30 to 90 days [17]. This process generates novel bioactive molecules, many of which are still unknown. Black garlic exhibits antiviral, antidiabetic, anti-obesity, anticancer, antimicrobial, antibacterial, anti-inflammatory, antihypertensive, and antioxidant activities, as well as cardioprotective and neuroprotective properties [17,19]. Black garlic contains up to 10-fold more total phenolic content (TPC) than raw garlic, with a significant increase in phenolic acids and flavonoid content during thermal processing [20]. The same profile has been reported in black giant garlic, even with higher PP concentration than common black garlic [21,22]. Black garlic production process increases from 6- to 46-fold the SAC content due to superior GGT activity [17,23], which depends on the manufacturing conditions, variety/species of garlic, agricultural practices, handling, and environmental conditions. As described for PPs, black giant garlic has also shown a higher SAC content than common black garlic [24]; thus, SAC and PP content could be considered proper chemical markers for black giant garlic elaboration. The determination of both bioactive molecules is not trivial when considering the complexity of black giant garlic as a matrix. Several efforts have been dedicated to developing reliable methods for SAC determination [25,26,27]. Advanced extraction techniques like ultrasound-assisted extraction (UAE) and microwave-assisted extraction (MAE), have important advantages over traditional extraction methods, including a significant reduction in energy consumption, solvent volume, and time consumption. They require lower sample amount and allow the use of green solvents. In particular, MAE is an environmentally friendly technology that works better with green polar solvents like water and ethanol and provides faster extractions because of the better mass transfer rate [28]. Optimization of MAE using a chemometric tool (Design of Experiment—DOE) is essential to enhance extraction yields. For this study, a response surface methodology based on a face-centered central composite design (CCD) was selected because of its high efficiency and reduced number of experiments. It is one of the most employed chemometric techniques for optimizing bioactive molecule extraction [29,30] and (bio)chemical reactions [31]. Bioactive molecule detection and identification is usually a highly time-consuming procedure; to shorten this process, hyphenation methods that couple analytical separation and bioassays have been developed/improved in the last decade. Effect-directed analysis (EDA) on high-performance thin layer chromatography (HPTLC) is a promising approach for discovering novel bioactive molecules, especially useful for complex matrices like black garlic. This technique combines chromatographic separation with an enzymatic bioassay in situ on the plate, enabling simultaneous separation and bioactivity assessment on a single platform [32,33]. Additionally, HPTLC allows in situ chemical derivatization to identify chemical groups [34] and direct coupling to mass spectrometry (MS) for preliminary structural identification [3,35]. The objective of the present work was to optimize by chemometric tools a green MAE of SAC and PPs present in black Chiloe’s giant garlic to detect and identify novel bioactive molecules with antioxidant and/or inhibitory activities over cyclooxygenase, α-glucosidase, and acetylcholinesterase enzymes.

## 2. Materials and Methods

### 2.1. Reagents, Chemicals, and Standards

Cyclooxygenase 2 human (EC 1.14.99.1), α-glucosidase from *Saccharomyces cerevisiae* (E.C. number 3.2.1.20), acetylcholinesterase from *Electrophorus electricus* (electric eel) (E.C. number 3.1.17), Folin–Ciocalteu Reagent (2N), sodium bicarbonate (≥99%), ninhydrin, 2,2-diphenyl-1-picrylhydrazyl (DPPH), arachidonic acid (≥98.5%), N,N,N′,N′-tetramethyl-p-phenylenediamine TMPD (≥98.5%), porcine hematin (≥90%), Tris–HCl, 1-naphthyl acetate, Fast Blue B salt (≥95%), bovine serum albumin (BSA), gallic acid (≥98%), and S-allyl-cysteine (≥98%) were purchased from Sigma (St. Louis, MO, USA). Additionally, 2-naphthyl-α-D-glucopyranoside (≥99%, substrate) was obtained from Glycosynth (Warrington, Cheshire, UK). Unless otherwise indicated, all solvents were p.a. ethanol, methanol, n-butanol, hydrochloric acid (37%), acetic acid, formic acid, water (MS grade), and acetonitrile (MS grade), purchased from Merck (Darmstadt, Germany). Positive control, diclofenac (OPKO, Lot # 220,803), and donepezil (HETERO, Lot # DNP20073A), were purchased from a local drugstore (Saludfarm, Chillán, Chile). Ultrapure water (18.2 MΩ cm) was produced using a PURIST Ultrapure Water system from RephiLe (Shanghai, China). The 96-well microplates were obtained from JET Biofil (Guangzhou, China).

### 2.2. Sample Preparation

All black Chiloe’s giant garlics were cultivated and elaborated on Chiloe Island (42°40′36″ S to 73°59′36″ W). Sample preparation was performed following the protocol reported by Peterssen-Fonseca et al. [10]. Briefly, all samples were first blanched for 10 min with hot water (ca. 90 °C) in a self-sealing plastic bag to inactive enzymes (alliinase and γ-glutamyl transpeptidase enzymes). Then, they were blended with a hand mixer and turned into a slurry, which was frozen at −20 °C for 48 h and freeze-dried at −55 °C for 24 h, using a Martin Christ (Osterode am Harz, Germany) Alpha 1–2 LD plus freeze dryer. The dried samples were ground into a fine powder using a mortar and stored at −20 °C until use.

### 2.3. Microwave-Assisted Extraction (MAE)

Extractions were performed using a Milestone (Sorisole, BG, Italy) Ethos X Microwave extractor. The operational conditions for all experimental runs were performed using a SK-15 rotor with contactless temperature and pressure sensors and a constant power of 500 W. Five minutes of initial heating and 5 min of final cooling. The most relevant MAE factors, i.e., extraction temperature (30–70 °C), ethanol percentage in water (0–40% *v*/*v*), and reaction time (3–12 min), were optimized using a face-centered CCD. Five hundred milligrams of the freeze-dried sample were loaded into a high-pressure microwave vessel, and 12 mL of the proper solvent mixture was added according to the experimental plan. After each extraction, the extracts were filtered through Whatman (Piscataway, NJ, USA) polyvinylidene difluoride (PVDF) 13 mm syringe filters (0.22 µm) and protected from light at −20 °C until further analysis.

### 2.4. Total (Poly)phenol Content (TPC)

TPC was performed using the method described by Carrasco-Sandoval et al. [36] with some modifications. Briefly, 15 µL of black garlic extract (or standard) was mixed with 100 µL of Folin–Ciocalteu reagent (0.2 N) and 100 µL of sodium bicarbonate (60 g/L) in a 96-well microplate. After 90 min (protected from light), the optical density (OD) was measured at 750 nm and 25 °C using a BioTek (Winooski, VT, USA) Epoch 2 microplate reader. Standard calibration was established using gallic acid (0.02 to 0.30 mg/mL) and results were expressed as mg of gallic acid equivalents (GAE) per 100 g of dry weight (DW).

### 2.5. Organosulfur Compound (OSC) Determination

OSCs were determined following the protocol N° F06A from CAMAG (Muttenz, Switzerland). Briefly, samples and SAC standard (0.1 mg /mL in 0.01 mol/L HCl) solutions were applied on an HPTLC silica gel 60 F_254_ plate using a CAMAG Automatic TLC Sampler 4 (ATS4) or semi-automatic TLC sample, Linomat 5, with the following settings for 10 × 10 cm and 20 × 20 cm plates: band length 5 mm, track distance 8.5 mm, dosage speed 150 nL/s, and first application *x*-axis and *y*-axis at 10 mm. Chromatography was performed in the CAMAG twin-through chamber up to a migration distance of 80 mm using a mobile phase composed of n-butanol: water: acetic acid: formic acid (28:8:9:2 *v*/*v*/*v*/*v*). After separation, the plate was dried at 60 °C for 10 min on a CAMAG TLC plate heater. Dried plates were derivatized by immersion for 3 s (3 cm/s) into a 0.3% *m*/*v* ninhydrin ethanolic solution using CAMAG immersion device 3. The plate was then heated for 20 min at 120 °C on a TLC plate heater. Detection was performed with CAMAG TLC in Vis-absorption mode at 520 nm with a slit dimension of 4.0 mm × 0.1 mm and a scanning speed of 20 mm/s. Instrument control and data acquisition and processing were performed using WinCATS 1.4.7 software.

### 2.6. EDA-HPTLC-Bioassay

Black Chiloe’s giant garlic extracts were applied to HPTLC plates using the same procedure and chromatographic separation described in Section 2.5. Before the bioassays, the plates were dried for 1 h at 60 °C to eliminate the acid present in the mobile phase. Positive controls were applied post-chromatography at 50 mm (*y*-axis). Based on the retention factor (*R*_F_), the bands of interest were located on a second HPTLC plate (or section) and identified as described in Section 2.7. For the **antioxidant assay,** the DPPH method was carried out using the conditions reported by López et al. [37] using caffeic acid (100 ng per band) as a positive control. HPTLC plate sections were immersed for 3 s (3 cm/s) into 0.5 mM (0.1% *m*/*v*) DPPH methanolic solution using CAMAG Immersion Device 3. After 30 min of incubation at room temperature in the dark, antioxidant molecules were observed as yellow bands on a purple background. **Cyclooxygenase (COX) inhibitory assay** was performed following the method established for Oyarzún et al. [38] using diclofenac (1 µg per band) as the positive control. All solutions were applied using a CAMAG Derivatizer. After a 10 min incubation in a humidity chamber at 37 °C, inhibitory bands appeared as colorless bands against a blue/purple background. **α-glucosidase (AG) inhibitory assay** was performed according to the conditions described by Galarce-Bustos et al. [35], using caffeic acid (100 ng per band) as the positive control. All solutions were applied using a CAMAG Derivatizer. After incubation in a humidity chamber at 37 °C for 20 min, a Fast Blue B salt (1 mg/mL) solution was sprayed onto the plate, and inhibitory molecules were observed as colorless bands against a purple/pink background. **Acetylcholinesterase (AChE) inhibitory assay** was performed following the method reported by Galarce-Bustos et al. [3], using donepezil (100 ng per band) as the positive control. After 20 min of incubation in a humidity chamber at 37 °C, a Fast Blue B salt solution (1 mg/mL) was sprayed onto the plate, and inhibitory molecules were observed as colorless bands against a pink/purple background.

### 2.7. Identification of Bioactive Molecules

Based on the retention factor (*R*_F_), all bands of interest were located on the second HPTLC plate or section (without bioassay reagents) and marked using a soft pencil inside of Spectroline (Melville, NY, USA) UV-cabinet. Bioactive molecules were identified in two steps. First, the bands of interest were directly eluted and analyzed by MS; and second, bands were eluted or scraped off to a micro-vial for liquid chromatography (LC) diode array (DAD)-MS analysis. For the first step, bands were eluted by means of the Advion Plate Express TLC/MS interface using methanol: 10 mM ammonium formate (19:1% *v*/*v*) as the elution solvent at a flow rate of 0.1 mL/min for 2 min to the electrospray ionization (ESI) source of Advion expression-L Compact MS and analyzed using the following conditions: capillary voltage (−2.5; 3.5 kV), nebulizing gas nitrogen (N_2_) 3 L/min, drying gas flow (N_2_) 10 L/min, DL temperature 200 °C, and block temperature 250 °C. Mass spectra were acquired in full scan mode (*m*/*z* 100–2000) applying positive and negative ionization. The plate background signals were subtracted for each analysis. Data were acquired through Advion Mass Express and processed using Data Express software version 5.1. For the second step, based on *R*_F_, all bands of interest were removed and extracted in methanol: water solution (7:3 *v*/*v*), filtered (0.22 µm), and injected for analysis into a Waters (Milford, MA, USA) Arc LC-system coupled to 2489 UV-Vis and QDa (single quadrupole) detectors. Chromatography was carried out using a binary mobile phase composed of water (A) and acetonitrile (B), both acidified with 0.1% *v*/*v* formic acid, following the method proposed by Liu et al. [39] on a Phenomenex (Torrance, CA, USA) Kinetex 3.5 µm XB-C_18_ core-shell column (150 mm × 4.6 mm, 100 Å) set at 30 °C. MS analysis was carried out using the following settings: ESI (+) capillary voltage of 0.8 kV, cone voltage of 10 V, probe temperature of 600 °C, source temperature of 120 °C in full scan mode (*m*/*z* 100–1200). To complete the identification, the sample was analyzed using the same chromatographic conditions in a Waters I-Class LC-system coupled to a 2998 PDA detector and an Ion Mobility (IMS)-High Resolution (HR) Mass Spectrometer (Synapt XS 32K). The mass spectrometer was set as follows: MS^e^ in continuum mode, ESI (+), capillary voltage of 0.8 kV, cone voltage of 20 V, scan speed of 0.3 s in resolution mode. The collision energy was set at 6 V for low energy and a ramp of 15–45 V for high energy (pseudo-MS/MS). For mass correction, a locked mass solution of leucine enkephalin was applied at intervals of 30 s. Before analysis, the mass detector was calibrated with sodium formate in the *m*/*z* range of 50–1200. Data were acquired and processed using Waters Masslynx 4.2 software.

### 2.8. Docking and Molecular Dynamics Studies

The three-dimensional structures of human cyclooxygenase-2 (COX-2), acetylcholinesterase (AChE), and α-glucosidase (AG) enzymes were retrieved from the Protein Data Bank with the following codes: 5KIR, 1DX6, and 5NN3 [40,41,42]. The 3D chemical structures of the isolated N-fructosylglutamylphenylalanine, N-fructosyl-glutamyl-S-(1-propenyl) cysteine, and harmane compounds were built and optimized in gas phase at the DFT level of theory using the B3LYP functional and the 6–31G(d,p) basis set, as implemented in the Gaussian 09 package of programs [43]. Prior to docking studies, the RESP charges for all compounds were calculated [44]. The docking studies were performed by means of AutoDock 4.2 [45], and the docking grid maps were calculated using AutoGrid4. The docking grids were centered on the catalytic residues defining a volume of 60 Å^3^ with a 0.375 Å grid spacing. The AutoTors option of AutoDockTools was used to define rotatable bonds. The genetic Lamarckian algorithm was used under the following conditions: population size 50, maximum number of evaluations 2,500,000, maximum number of generations 27,000, rate of mutation 0.02, and rate of crossover 0.08. The calculations were performed with dielectric as the default setting. The most stable conformations for each compound were chosen according to the best docking score, the population of the conformation and the activity documented in this study. The previously obtained complexes were inserted into a TIP3P water box using the CHARMM-GUI server [46]. The ionic force considered the addition of NaCl at 0.15 mol/L. Titratable residues were kept in their dominant protonation state at pH 7.0. The FF19SB forcefield [47] was selected for molecular dynamics simulations by means of AMBER18 suite of programs [48]. The protocol considered an initial minimization of 5000 steps using the conjugate gradient method, followed by 2500 steps of steepest descent. A heating step was then conducted from 0 to 310 K in an NPT assembly for 100 ps using a harmonic restraint of 10.0 kcal/mol/Å^2^ for protein’s atoms. Subsequently, the system was subjected to a 1 ns equilibration step without restrictions, allowing the system to reach equilibrium. Additionally, production runs of 100 ns were conducted to calculate the binding energy and the binding free energy (ΔG) using MMPBSA.py a Phyton script part of AmberTools19 [48,49].

### 2.9. Statistical Analysis

Data were evaluated using descriptive statistics [mean, standard deviation (SD), and relative standard deviation (RSD)]. Calibrations were established by applying linear and polynomial regression models. Statistical tests were performed at a significance level of (α) 0.05 using Prism 10 software (GraphPad, San Diego, CA, USA). The response surface methodology based on central composite design (CCD) was prepared and analyzed using StatGraphics Centurion XV version 15.1.02 (Rockville, MD, USA).

## 3. Results and Discussion

### 3.1. Optimization of MAE Conditions for PP and OSC Extraction

Some studies have discussed the optimization of black garlic and black giant garlic production parameters, focusing on achieving higher bioactive molecule content such as SAC and PPs [50,51,52]. However, information concerning the optimization of the extraction process is still limited. MAE was selected to perform PP and OSC extraction from black Chiloe’s giant garlic because it presents important advantages over conventional and other advanced extraction techniques. This extraction process offers fast and efficient extraction with high yields using reduced solvent volumes and low sample amounts, with the possibility of using green solvents such as ethanol and water. According to preliminary assays and earlier studies on black garlic, a response surface methodology based on a face-centered CCD was established with three independent factors, i.e., temperature (30 to 90 °C), ethanol percentage in water (0 to 40%), and extraction time (3 to 12 min) [53,54]. The experimental plan consisted of 16 runs, including two central points (Table 1), each performed in triplicate (*n* = 3) in random order to minimize the effects of uncontrolled factors. The responses/variables studied were the TPC and SAC extraction yields, as described in Section 2.4 and Section 2.5.

**Figure 1:**
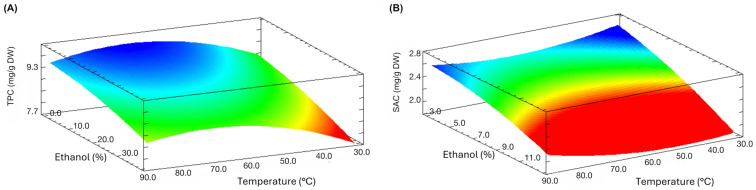
Response surface plots of MAE of PPs and SAC from black Chiloe’s giant garlic showing the effect of independent variables on extraction yields. (**A**) TPC plot at 12 min; (**B**) SAC content plot at 40% *v*/*v* ethanol.

Through response surface optimization (Figure 1), the optimal MAE conditions were established for SAC and TPC (Table 2). According to the data, TPC and SAC optimal extraction conditions were accomplished at 67 °C for 12 min and 0% *v*/*v* ethanol, and 30 °C for 3 min and 40% *v*/*v* ethanol, respectively. Both equations (Figure S1, Supplementary Materials) shown a non-significant lack of fit (*p* > 0.05) obtaining optimal TPC and SAC concentrations of 9.19 ± 0.18 mg GAE/g DW and 2.55 ± 0.10 mg SAC/g DW, with an error of 6.3% and 4.2%, respectively, showing proper fitting. These optimal conditions could be explained by the specific nature of garlic. PPs are typically found linked to bulb cell walls and/or forming glycosides; in both cases, MAE exerts a disruptive effect, facilitating its extraction [55]. Additionally, in the case of black garlic, the mild Maillard reaction increases the dielectric constant, accelerating the extraction process in the presence of water [56].

Compared with conventional extraction techniques, maceration extraction of PPs from raw and black garlics was optimized using a response surface methodology. The optimal extraction conditions were 47 °C, 50% *v*/*v* ethanol for 6 h, and 90 °C, 55% *v*/*v* ethanol for 9.8 h, respectively [54,57]. Both optimal conditions required much higher extraction times (9.8 h) than the proposed MAE method (12 min, 98% lower). Kim et al. [21] reported a TPC value of 9.95 ± 0.06 mg GAE/g in black giant garlic, similar to the value reported in the present work for black Chiloe’s giant garlic (9.19 ± 0.18 mg GAE/g DW). However, the TPC value was obtained with a time-consuming extraction procedure (72 h, 3 consecutive extractions of 24 h each), which was much more extensive than that reported in the present work (12 min).

Compared with previous reports, the main advantage of the MAE-based method for SAC extraction was the time required for proper extraction, needing only 3 min [58]. The solvent composition is consistent with that of earlier studies [24,58]. The SAC concentration found in black Chiloe’s giant garlic is 3- to 20-fold higher than that described for common black garlic and 12-fold greater than that of Korean black giant garlic after a 72 h extraction process [24,58]. These findings demonstrate the importance of optimizing the extraction method and show the extraordinarily high SAC content in black Chiloe’s giant garlic.

### 3.2. Identification of Bioactive Molecules in Black Chiloe’s Giant Garlic

**Figure 2:**
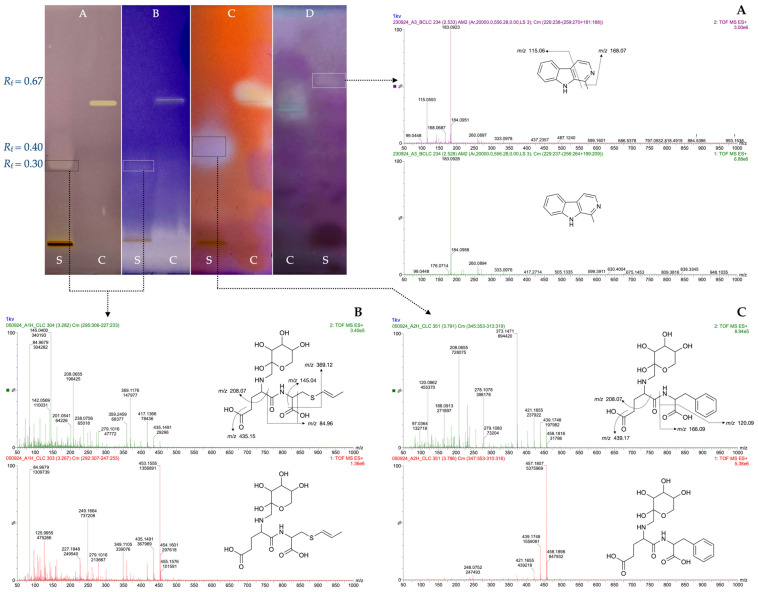
HPTLC chromatogram of optimized black Chiloe’s giant garlic extracts (S) and controls (C) after derivatization with DPPH (A), and bioassays to detect inhibitors of COX-2 (B), AG (C), and AChE (D), which were identified by mass spectra and fragmentation pattern as harmane, parent ion at *m*/*z* 183.0928 [M+H]^+^ (**A**); N-fructosyl-glutamyl-S-(1-propenyl) cysteine, parent ion at *m*/*z* 453.1555 [M+H]^+^(**B**) and N-fructosyl-glutamylphenylalanine, parent ion at *m*/*z* 457.1822 [M+H]^+^ (**C**).

From optimized black Chiloe’s giant garlic extracts, bioactive molecules with antioxidant and inhibitory activities over COX-2, AG, and AChE were detected and identified by HPTLC-Bioassay and LC-HRMS, as described in Section 2.6. The identification process included *R*_F_ analysis, chemical identification, mass spectrometry, fragmentation analysis, and mass error (Table 3). As shown in Figure 2, the band at *R*_F_ 0.67 showed AChE inhibitory activity with a *m*/*z* of 183.0928 [M+H]^+^ and main fragments at *m*/*z* 115.0533 (Figure 2A) corresponding to the indole alkaloid derivative methyl-β-Carboline, most likely harmane, which is a tryptophan-derived Maillard product previously reported in black garlic [59]. Harmane possesses a broad spectrum of bioactivities, including AChE inhibition [60]. The band at *R*_F_ (0.3) exhibited both antioxidant and COX-2 inhibitory activities. MS analysis revealed a main compound with *m*/*z* 453.1555 [M+H]^+^ and fragments at *m*/*z* 369.1176, 208.1655, and 145.0400, which was identified as N-fructosyl glutamyl-S-(1-propenyl) cysteine (Figure 2B). This compound retains both biological properties of glutamyl-S-(1-propenyl) cysteine [38]. Similarly, a band at *R*_F_ 0.4 showed α-glucosidase inhibitory activity (Figure 2) with an *m*/*z* value of 457.1822 [M+H]^+^ and main fragments at *m*/*z* 373.1471 and 208.0655 (Figure 2C). This compound was identified as N-fructosyl glutamyl-S-phenylalanine, which is more than 2000 times more abundant in black garlic than in raw garlic [59]. Both compounds are byproducts of the early stage of the Maillard reaction [59], and these data could be useful as biomarkers for black garlic elaboration. Glycation of these glutamyl peptides seems to play an important role in the pharmacokinetic profile and proteolytic stability and could also be structurally related to anti-inflammatory and antidiabetic activity [61]. Since giant garlic has considerable higher glutamyl peptides content than common garlic [13], compounds such as N-fructosyl glutamyl-S-(1-Propenyl) cysteine and N-fructosyl glutamylphenylalanine and others bioactive Millard metabolites is expected to be produced in relevant amounts in black Chiloe’s giant garlic. Since no commercial standards of N-fructosyl glutamyl-S-(1-propenyl) cysteine and N-fructosyl glutamylphenylalanine were available, the possible interactions were assessed by docking studies.

### 3.3. Docking and Molecular Dynamics Analysis

In AG, a carbohydrate-hydrolase enzyme that releases a single alpha-glucose molecule from non-reducing α-glucose residues (1→4-linked), Trp-516 and Asp-518 are identified as critical residues for its catalytic functionality. The molecule N-fructosyl glutamylphenylalanine forms hydrogen bonds between the N-fructosyl hydroxyl groups and Asp-518 of the active site residue as illustrated in Figure S2 (yellow color, Supplementary Materials). Similar to acarbose, AG inhibitor drug, the sugar structure stays in the same cavity bound to Asp-518 with 5 hydrogen bond donor groups and a hydrogen bond acceptor from the NH group [62]. The Trp-516 structure is implicated in the formation of a hydrophobic region, wherein the N-fructosyl moiety remains (Figure S2, Supplementary Materials). The COX-2 active site contains several key residues such as Arg-120 involved in the catalysis and Tyr-355, which have been observed to form a hydrogen bond with arachidonic acid. Tyr-385 plays a pivotal role in the transformation of polyunsaturated fatty acids (PUFA). Glu-524 constitutes a component of the constriction separating the active site from the membrane binding domain. Val-523 is an integral part of the binding cavity. Other amino acids in the active site include Val-434, Leu-503, and Arg-513. The docking results indicate that upon binding, the N-fructosyl-glutamyl-S-(1-propenyl) cysteine compound could interact with active site residues as shown in Figure S3 (Supplementary Materials). In the case of AChE, the active site contains the catalytic triad of amino acids, namely Ser-226, Glu-327, and His-440. These amino acids are in a deep, narrow gorge at the bottom of the enzyme. The active site is also lined with other amino acids that aid in substrate binding and stabilization. Trp-86 is an aromatic amino acid that binds to the choline segment of the substrate. Phe-295, Phe-297, and Trp-236 form an acyl-binding pocket that stabilizes the acetyl group of acetylcholine. Also, Gly-121, Gly-122, and Ala-204 form an oxyanionic gap that stabilizes the transition state of the substrate. Other residues such as Tyr-70, Tyr-121, and Trp-279 form the peripheral anionic site (PAS). According to docking results observed in Figure S4 (Supplementary Materials), the Harmane molecule is found interacting mainly with His-440 in the active site (yellow).

It is important to note that docking studies are not able to indicate whether a molecule will act as a substrate or inhibitor of an enzyme; however, they can provide an account of which residues these molecules may interact with. In this regard, to elucidate the dynamic characteristics of these molecular interactions, each docked complex was subjected to molecular dynamics simulations, followed by binding free energy calculations (ΔG). The complexes with the most favorable binding free energy were subjected to further analysis. All dynamics simulations revealed the same key interactions as obtained by docking campaign. Moreover, the reported IC50 was compared with ΔG values, observing reasonable correlations according to the negative total binding free energies reported (Table 4). Thus, the identified compounds seem to be great prospects, especially fructosyl glutamyl peptides. The energy values reveal favorable protein–protein complex in pure water, but these results do not equal the real binding free energy since there is no estimation of the (unfavorable) entropy contribution to binding. The Generalized Born approach gives lower binding energies, suggesting that all enzyme complexes included in this study are in a favorable bound state. Thus, given the established role of molecules as enzyme inhibitors, as confirmed by HPTLC-Bioassay, the latter conclusion was supported by dynamic simulations.

## 4. Conclusions

To the best of our knowledge, the present work reports for the first time the optimization of the MAE of PPs and SAC from black Chiloe’s giant garlic. This optimized technique allowed the obtention of extracts with higher SAC concentrations than previous reports (3- to 20-fold high) using reduced extraction times (3 min for SAC and 12 min for PPs), which were at least 98% lower than previous reports. These results demonstrated the relevance of advanced extraction technologies like MAE, especially using green solvents (water and ethanol), which are emerging as promising techniques for the quality control of black garlic elaboration. These optimized extracts exhibited multiple bioactivities, some of which were described for the first time in black Chiloe’s giant garlic. A key point of this work was the direct identification of bioactive molecules through EDA-HPTLC-Bioassay-MS, reporting three novel associations between molecules and biological activity. Thanks to this approach, it was possible to advance the general approximation of bioactive extracts without identifying the molecule (s) responsible for the effect. The bioactive molecules identified showed antioxidant and/or inhibitory activities on COX-2, AG, and AChE, which established the therapeutic potential of this type of garlic as a food and/or functional ingredient to reduce the risk of CNCD. Docking and molecular dynamics analysis showed proper ΔG values compared with commercial inhibitors; thus, the identified compounds in black Chiloe’s giant garlic seem to be great prospects specially fructosyl glutamyl peptides.

## Figures and Tables

**Table 1:** Experimental runs (*n* = 3) for central composite design showing independent variables and experimental data for PP and SAC yields (mean ± SD).

Runs	T° (°C)	Ethanol (%)	Time (min)	TPC (mg GAE/g DW)	S-Allyl-Cysteine (mg/g DW)
1	30	40	3.0	8.21 ± 0.41	2.87 ± 0.24
2	90	20	7.5	8.72 ± 0.56	2.87 ± 0.01
3	30	0	12.0	9.01 ± 0.30	2.64 ± 0.01
4	60	20	7.5	9.42 ± 0.37	2.25 ± 0.03
5	30	0	3.0	8.32 ± 0.52	2.70 ± 0.15
6	60	40	7.5	8.68 ± 0.67	2.61 ± 0.11
7	90	0	3.0	8.52 ± 0.31	2.45 ± 0.05
8	60	20	3.0	9.83 ± 0.66	2.43 ± 0.08
9	90	40	3.0	8.76 ± 0.57	2.19 ± 0.17
10	90	40	12.0	8.85 ± 0.23	2.16 ± 0.24
11	30	40	12.0	7.93 ± 0.56	1.84 ± 0.11
12	60	20	12.0	8.64 ± 0.40	1.97 ± 0.09
13	30	20	7.5	8.61 ± 0.49	1.94 ± 0.17
14	90	0	12.0	9.69 ± 0.44	2.12 ± 0.20
15	60	20	7.5	9.79 ± 0.43	2.06 ± 0.02
16	60	0	7.5	9.53 ± 0.39	2.05 ± 0.16

**Table 2:** Optimized conditions for MAE of SAC and PPs present in black Chiloe’s giant garlic.

	TPC	SAC
Temperature (30–90 °C)	67	30
Ethanol percentage (0–40%)	0	40
Time (3–12 min)	12	3
Predicted yields (mg/g)	9.81	2.66
Experimental yield (mg/g)	9.19 ± 0.18	2.55 ± 0.10
Percentage of error (%)	6.3	4.2
Lack of fit (*p*-value)	0.36	0.22

**Table 3:** Bioactive molecules present in black Chiloe’s giant garlic.

Band (*R*_F_)	Bioactivity *	Bioactive Molecule	Molecular Formula	Observed *m*/*z* (u)	Theorical *m*/*z* (u)	Mass Error (ppm)	Fragments *m*/*z* (u)
0.30	Antioxidant and COXi	N-Fructosyl Glutamyl-S-(1-Propenyl) cysteine	C_17_H_28_N_2_O_10_S	453.1555	453.1543	2.65	369.1176 208.1655 145.0400
0.40	AGi;	N-Fructosyl Glutamylphenylalanine	C_20_H_28_N_2_O_10_	457.1807	457.1822	−3.28	373.1471 208.0655 166.0913
0.67	AChEi	Methyl-β-Carboline	C_12_H_10_N_2_	183.0928	183.0922	3.27	115.0553

* COXi, AGi, and AChEi correspond to cyclooxygenase, α-glucosidase, acetylcholinesterase enzyme inhibitors.

**Table 4:** Calculated binding energies of enzyme inhibitors present in black Chiloe’s giant garlic compared with commercial ones.

Complex		Molecular Dynamics		IC_50_ Values	Ref.
Enzyme	Substrate	ΔG_MMGBSA_ (kcal/mol)	ΔG_MMPBSA_ (kcal/mol)		
AG	Acarbose	−8.2	−6.4	93.63 μM	[63]
	N-fructosyl glutamylphenylalanine	−27.5	−11.8	-	*
AChE	Donepezil	−34.5	−19.2	6.7 nM	[64]
	Harmane	−11.2	−7.3		*
COX-2	Diclofenac	−33.7	25.3	1.3 nM	[65]
	N-Fructosyl Glutamyl-S-(1-Propenyl) cysteine	−32.4	−24.4	-	*

* This work.

## Data Availability

Data is available on request from corresponding authors.

## Supplementary Materials

The following supporting information can be downloaded at https://www.mdpi.com/article/10.3390/antiox14080913/s1, Figure S1: Mathematical models for TPC (mg GAE/g DW) and SAC (mg/g DW); Figure S2: N-fructosyl-glutamylphenylalanine (cyan) forms hydrogen bonds between the N-fructosyl hydroxyl groups and the active site residue Asp-518 (yellow); Figure S3: Interaction of N-fructosyl-glutamyl-S-(1-propenyl) cysteine (cyan) with active site residues Arg-120 and with Tyr-385 (yellow); Figure S4: Interaction of Harmane molecule (cyan) with the His-440 residue (yellow). At this position, Harmane remains shielded by several aromatic residues.

## References

[1] WHO (2022). World Health Statistics 2022: Monitoring Health for the SDGs, Sustainable Development Goals.

[2] Singh R.B., Watanabe S., Isaza A.A. (2021). Functional Foods and Nutraceuticals in Metabolic and Non-communicable Diseases. Functional Foods and Nutraceuticals in Metabolic and Non-Communicable Diseases.

[3] Galarce-Bustos O., Pavon J., Henriquez-Aedo K., Aranda M. (2019). Detection and identification of acetylcholinesterase inhibitors in *Annona cherimola* Mill. by effect-directed analysis using thin-layer chromatography-bioassay-mass spectrometry. Phytochem. Anal..

[4] El-Saber Batiha G., Magdy Beshbishy A., Wasef L.G., Elewa Y.H.A., Al-Sagan A.A., Abd El-Hack M.E., Taha A.E., Abd-Elhakim Y.M., Prasad Devkota H. (2020). Chemical Constituents and Pharmacological Activities of Garlic (*Allium sativum* L.): A Review. Nutrients.

[5] Najda A., Błaszczyk L., Winiarczyk K., Dyduch J., Tchórzewska D. (2016). Comparative studies of nutritional and health-enhancing properties in the “garlic-like” plant *Allium ampeloprasum* var. ampeloprasum (GHG-L) and A. sativum. Sci. Hortic..

[6] Iciek M., Kwiecień I., Włodek L. (2009). Biological properties of garlic and garlic-derived organosulfur compounds. Environ. Mol. Mutagen..

[7] Lu X., Ross C.F., Powers J.R., Aston D.E., Rasco B.A. (2011). Determination of total phenolic content and antioxidant activity of garlic (*Allium sativum*) and elephant garlic (*Allium ampeloprasum*) by attenuated total reflectance-fourier transformed infrared spectroscopy. J. Agric. Food Chem..

[8] Wong S.P., Leong L.P., William Koh J.H. (2006). Antioxidant activities of aqueous extracts of selected plants. Food Chem..

[9] Figliuolo G., Di Stefano D. (2007). Is single bulb producing garlic *Allium sativum* or *Allium ampeloprasum*?. Sci. Hortic..

[10] Peterssen-Fonseca D., Henríquez-Aedo K., Carrasco-Sandoval J., Cañumir-Veas J., Herrero M., Aranda M. (2021). Chemometric optimisation of pressurised liquid extraction for the determination of alliin and S-allyl-cysteine in giant garlic (*Allium ampeloprasum* L.) by liquid chromatography tandem mass spectrometry. Phytochem. Anal..

[11] Hirschegger P., Galmarini C., Bohanec B. (2006). Characterization of a novel form of fertile great headed garlic (*Allium* sp.). Plant Breed..

[12] FAO (2009). Globally Important Agricultural Heritage Systems (GIAHS).

[13] Kim S., Kim D.B., Jin W., Park J., Yoon W., Lee Y., Kim S., Lee S., Kim S., Lee O.H. (2018). Comparative studies of bioactive organosulphur compounds and antioxidant activities in garlic (*Allium sativum* L.), elephant garlic (*Allium ampeloprasum* L.) and onion (*Allium cepa* L.). Nat. Prod. Res..

[14] Ceccanti C., Rocchetti G., Lucini L., Giuberti G., Landi M., Biagiotti S., Guidi L. (2021). Comparative phytochemical profile of the elephant garlic (*Allium ampeloprasum* var. holmense) and the common garlic (Allium sativum) from the Val di Chiana area (Tuscany, Italy) before and after in vitro gastrointestinal digestion. Food Chem..

[15] Yudhistira B., Punthi F., Lin J.-A., Syahrullah Sulaimana A., Chang C.-K., Hsieh C.-W., Chang-Wei Hsieh C. (2022). S-Allyl cysteine in garlic (*Allium sativum*): Formation, biofunction, and resistance to food processing for value-added product development. Compr. Rev. Food Sci. Food Saf..

[16] Park T., Oh J.H., Lee J.H., Park S.C., Jang Y.P., Lee Y. (2017). Oral Administration of (S)-Allyl-l-Cysteine and Aged Garlic Extract to Rats: Determination of Metabolites and Their Pharmacokinetics. Planta Med..

[17] Bae S.E., Cho S.Y., Won Y.D., Lee S.H., Park H.J. (2014). Changes in S-allyl cysteine contents and physicochemical properties of black garlic during heat treatment. LWT—Food Sci. Technol..

[18] Valls R.M., Companys J., Calderón-Pérez L., Salamanca P., Pla-Pagà L., Sandoval-Ramírez B.A., Bueno A., Puzo J., Crescenti A., Bas J.M.d. (2022). Effects of an Optimized Aged Garlic Extract on Cardiovascular Disease Risk Factors in Moderate Hypercholesterolemic Subjects: A Randomized, Crossover, Double-Blind, Sustainedand Controlled Study. Nutrients.

[19] Javed M., Ahmed W. (2022). Black garlic: A review of its biological significance. J. Food Biochem..

[20] Kim J.-S., Kang O.-J., Gweon O.-C. (2013). Comparison of phenolic acids and flavonoids in black garlic at different thermal processing steps. J. Funct. Foods.

[21] Kim D., Kim K.-H., Yook H.-S. (2019). Comparison of antioxidant activity between black elephant garlic (*Allium ampeloprasum*) and black normal garlic (*Allium sativum* L.). J. Korean Soc. Food Sci. Nutr..

[22] Nam S.-H., Han Y.-S., Sim K.-H., Yang S.-O., Kim M.-H. (2023). Changes in the Physicochemical Properties, Antioxidant Activity and Metabolite Analysis of Black Elephant Garlic (*Allium ampeloprasum* L.) during Aging Period. Foods.

[23] Ai T.T., Huong N.T. (2018). Research on the production of black garlic juice. Int. J. Pharm. Sci. Invent..

[24] Kim D., Kim K.-H., Yook H.-S. (2015). Analysis of active components of giant black garlic. J. Korean Soc. Food Sci. Nutr..

[25] Bae S.E., Cho S.Y., Won Y.D., Lee S.H., Park H.J. (2012). A comparative study of the different analytical methods for analysis of S-allyl cysteine in black garlic by HPLC. LWT—Food Sci. Technol..

[26] Malaphong C., Tangwanitchakul A., Boriboon S., Tangtreamjitmun N. (2022). A simple and rapid HPLC method for determination of S-allyl-L-cystein and its use in quality control of black garlic samples. LWT—Food Sci. Technol..

[27] Tho D.C.M.V., Thao H.P., Tuan N.D., Zemann A. (2019). Quantitative analysis of S-Allylcysteine in black garlic via Ultra-High-Performance Liquid Chromatography-Tandem Mass Spectrometry. Syst. Rev. Pharm..

[28] Zhang Q.-W., Lin L.-G., Ye W.-C. (2018). Techniques for extraction and isolation of natural products: A comprehensive review. Chin. Med..

[29] Carrasco-Sandoval J., Rebolledo P., Peterssen-Fonseca D., Fischer S., Wilckens R., Aranda M., Henríquez-Aedo K. (2021). A fast and selective method to determine phenolic compounds in quinoa (*Chenopodium quinoa* Will) seeds applying ultrasound-assisted extraction and high-performance liquid chromatography. Chem. Pap..

[30] Galarce-Bustos O., Fernandez-Ponce M.T., Montes A., Pereyra C., Casas L., Mantell C., Aranda M. (2020). Usage of supercritical fluid techniques to obtain bioactive alkaloid-rich extracts from cherimoya peel and leaves: Extract profiles and their correlation with antioxidant properties and acetylcholinesterase and alpha-glucosidase inhibitory activities. Food Funct..

[31] Galarce-Bustos O., Novoa L., Pavon-Perez J., Henriquez-Aedo K., Aranda M. (2019). Chemometric Optimization of QuEChERS Extraction Method for Polyphenol Determination in Beers by Liquid Chromatography with Ultraviolet Detection. Food Anal. Methods.

[32] Morlock G.E., Heil J., Bardot V., Lenoir L., Cotte C., Dubourdeaux M. (2021). Effect-Directed Profiling of 17 Different Fortified Plant Extracts by High-Performance Thin-Layer Chromatography Combined with Six Planar Assays and High-Resolution Mass Spectrometry. Molecules.

[33] Morlock G.E., Belay A., Heil J., Mehl A., Borck H. (2022). Effect-Directed Profiling of Monofloral Honeys from Ethiopia by High-Performance Thin-Layer Chromatography and High-Resolution Mass Spectrometry. Molecules.

[34] Aranda M., Vega M.H., Villegas R.F. (2005). Routine method for quantification of starch by planar chromatography (HPTLC). JPC J. Planar Chromatogr.—Mod. TLC.

[35] Galarce-Bustos O., Pavon-Perez J., Henriquez-Aedo K., Aranda M. (2019). An improved method for a fast screening of alpha-glucosidase inhibitors in cherimoya fruit (*Annona cherimola* Mill.) applying effect-directed analysis via high-performance thin-layer chromatography-bioassay-mass spectrometry. J. Chromatogr. A.

[36] Carrasco-Sandoval J., Falcó I., Sánchez G., Fabra M.J., López-Rubio A., Rodriguez A., Henríquez-Aedo K., Aranda M. (2022). Multivariable optimization of ultrasound-assisted extraction for the determination of phenolic and antioxidants compounds from arrayan (*Luma apiculata* (DC.) Burret) leaves by microplate-based methods and mass spectrometry. J. Appl. Res. Med. Aromat. Plants.

[37] Lopez K., Espinoza-Bello A., Carrasco J., Peña-Farfal C., Aranda M., Henriquez-Aedo K. (2023). Multivariate optimization of microwave-assisted enzyme digestion of α-casein for generation of bioactive peptides. J. Chil. Chem. Soc..

[38] Oyarzún P., Carrasco J., Peterssen D., Tereucan G., Aranda M., Henríquez-Aedo K. (2023). A high throughput method for detection of cyclooxygenase-2 enzyme inhibitors by effect-directed analysis applying high performance thin layer chromatography-bioassay-mass spectrometry. J. Chromatogr. A.

[39] Liu P., Weng R., Sheng X., Wang X., Zhang W., Qian Y., Qiu J. (2020). Profiling of organosulfur compounds and amino acids in garlic from different regions of China. Food Chem..

[40] Roig-Zamboni V., Cobucci-Ponzano B., Iacono R., Ferrara M.C., Germany S., Bourne Y., Parenti G., Moracci M., Sulzenbacher G. (2017). Structure of human lysosomal acid α-glucosidase–a guide for the treatment of Pompe disease. Nat. Commun..

[41] Orlando B.J., Malkowski M.G. (2016). Crystal structure of rofecoxib bound to human cyclooxygenase-2. Acta Crystallgr. Sect. F.

[42] Greenblatt H.M., Kryger G., Lewis T., Silman I., Sussman J.L. (1999). Structure of acetylcholinesterase complexed with (−)-galanthamine at 2.3 Å resolution. FEBS Lett..

[43] Frisch M.J., Trucks G.W., Schlegel H.B., Scuseria G.E., Robb M.A., Cheeseman J.R., Scalmania G., Barone V., Petersson G.A., Nakatsuji H. (2016). Gaussian 09, Revision A.02.

[44] Vanquelef E., Simon S., Marquant G., Garcia E., Klimerak G., Delepine J.C., Cieplak P., Dupradeau F.Y. (2011). RED Server: A web service for deriving RESP and ESP charges and building force field libraries for new molecules and molecular fragments. Nucleic Acids Res..

[45] Morris G.M., Huey R., Lindstrom W., Sanner M.F., Belew R.K., Goodsell D.S., Olson A.J. (2009). AutoDock4 and AutoDockTools4: Automated docking with selective receptor flexibility. J. Comput. Chem..

[46] Jo S., Kim T., Iyer V.G., Im W. (2008). CHARMM-GUI: A web-based graphical user interface for CHARMM. J. Comput. Chem..

[47] Tian C., Kasavajhala K., Belfon K.A.A., Raguette L., Huang H., Migues A.N., Bickel J., Wang Y., Pincay J., Wu Q. (2020). ff19SB: Amino-Acid-Specific Protein Backbone Parameters Trained against Quantum Mechanics Energy Surfaces in Solution. J. Chem. Theory Comput..

[48] Case D.A., Cheatham T.E., Darden T., Gohlke H., Luo R., Merz K.M., Onufriev A., Simmerling C., Wang B., Woods R.J. (2005). The Amber biomolecular simulation programs. J. Comput. Chem..

[49] Miller B.R., McGee T.D., Swails J.M., Homeyer N., Gohlke H., Roitberg A.E. (2012). MMPBSA.py: An Efficient Program for End-State Free Energy Calculations. J. Chem. Theory Comput..

[50] Sasmaz H.K., Kadiroglu P., Adal E., Sevindik O., Aksay O., Erkin O.C., Selli S., Kelebek H. (2023). Optimization of black garlic production parameters using response surface methodology: Assessment and characterization of bioactive properties. J. Appl. Res. Med. Aromat. Plants.

[51] Chang T.C., Jang H.D. (2021). Optimization of Aging Time for Improved Antioxidant Activity and Bacteriostatic Capacity of Fresh and Black Garlic. Appl. Sci..

[52] Chang W.-C., Lin W.-C., Wu S.-C. (2023). Optimization of the Black Garlic Processing Method and Development of Black Garlic Jam Using High-Pressure Processing. Foods.

[53] Pakakaew P., Phimolsiripol Y., Taesuwan S., Kumphune S., Klangpetch W., Utama-ang N. (2022). The shortest innovative process for enhancing the S-allylcysteine content and antioxidant activity of black and golden garlic. Sci. Rep..

[54] Kang J.-R., Lee S.-J., Kwon H.-J., Kwon M.-H., Sung N.-J. (2012). Establishment of Extraction Conditions for the Optimization of the Black Garlic Antioxidant Activity Using the Response Surface Methodology. Korean J. Food Preserv..

[55] Vinatoru M., Mason T.J., Calinescu I. (2017). Ultrasonically assisted extraction (UAE) and microwave assisted extraction (MAE) of functional compounds from plant materials. TrAC Trends Anal. Chem..

[56] Sipahioglu O., Barringer S.A. (2003). Dielectric properties of vegetables and fruits as a function of temperature, ash, and moisture content. J. Food Sci..

[57] Anne R., Nithyanandam R. (2016). Optimization of extraction of bioactive compounds from medicinal herbs using response surface methodology. Int. Proc. Chem. Biol. Environ. Eng..

[58] Manoonphol K., Suttisansanee U., Promkum C., Butryee C. (2023). Effect of Thermal Processes on S-Allyl Cysteine Content in Black Garlic. Foods.

[59] Chang W.C.-W., Chen Y.-T., Chen H.-J., Hsieh C.-W., Liao P.-C. (2020). Comparative UHPLC-Q-Orbitrap HRMS-Based Metabolomics Unveils Biochemical Changes of Black Garlic during Aging Process. J. Agric. Food Chem..

[60] Nikam T.D., Nitnaware K.M., Ahire M.L., Ramawat K.G., Mérillon J.-M. (2013). Alkaloids derived from tryptophan: Harmine and related alkaloids. Natural Products: Phytochemistry, Botany and Metabolism of Alkaloids, Phenolics and Terpenes.

[61] Apostol C.R., Hay M., Polt R. (2020). Glycopeptide drugs: A pharmacological dimension between “Small Molecules” and “Biologics”. Peptides.

[62] Amini F., Abbas K.I., Ghasemi J.B. (2025). Molecular modeling approach in design of new scaffold of α-glucosidase inhibitor as antidiabetic drug. Biochem. Biophys. Rep..

[63] Feng Y., Nan H., Zhou H., Xi P., Li B. (2022). Mechanism of inhibition of α-glucosidase activity by bavachalcone. Food Sci. Technol..

[64] Makarian M., Gonzalez M., Salvador S.M., Lorzadeh S., Hudson P.K., Pecic S. (2022). Synthesis, kinetic evaluation and molecular docking studies of donepezil-based acetylcholinesterase inhibitors. J. Mol. Struct..

[65] Riendeau D., Percival M.D., Boyce S., Brideau C., Charleson S., Cromlish W., Ethier D., Evans J., Falgueyret J.P., Ford-Hutchinson A.W. (1997). Biochemical and pharmacological profile of a tetrasubstituted furanone as a highly selective COX-2 inhibitor. Br. J. Pharmacol..

