# Supplementary material for: Identification of Novel Bioactive Molecules in Black Chiloe’s Giant Garlic (Allium ampeloprasum L.) by Green Microwave-Assisted Extraction and Effect-Directed Analysis Using High-Performance Thin Layer Chromatography-Bioassay and Mass Spectrometry
Source: Antioxidants (Basel). 2025 Jul 25;14(8):913. doi: 10.3390/antiox14080913 (PMC12383059; doi:10.3390/antiox14080913)
Supplement: Supplementary file 1 [file antioxidants-14-00913-s001.zip › antioxidants-3527008-supplementary.pdf]

Supplementary Materials

Article

Identification of Novel Bioactive Molecules in Black Chiloe's Giant Garlic (*Allium ampeloprasum* L.) by Green Microwave-Assisted Extraction and Effect-Directed Analysis Using High-Performance Thin Layer Chromatography-Bioassay and Mass Spectrometry

Joaquín Fernández-Martínez[1], David Arráez-Román[2], Darlene Peterssen[3], Gerald Zapata[4], Karem Henríquez-Aedo[5*], Mario Aranda[1*]

1 Food and Drug Research Laboratory, Faculty of Chemistry and Pharmacy, Pontificia Universidad Católica de Chile, Santiago, Chile.; jefernandez2@uc.cl; mario.aranda@uc.cl
2 Department of Analytical Chemistry, Universidad de Granada, Granada, Spain. darraez@ugr.es
3 Department of Biological and Chemical Sciences, Faculty of Sciences, Universidad San Sebastián, Concepción, Chile. darlene.peterssen@uss.cl
4 Center of Molecular Modelling, Biophysics and Bioinformatics. Faculty of Chemistry and Pharmaceutical Sciences. Universidad de Chile, Santiago. gzapata@uchile.cl
5 Laboratory of Food Biotechnology and Genetics, Department of Basic Sciences, Faculty of Sciences, Universidad del Bío-Bío, Chillán, Chile. kahenriquez@ubiobio.cl

* Correspondence: K.H-A.: kahenriquez@ubiobio.cl; M.A.: mario.aranda@uc.cl

Gallic Acid = 6.53277 + 0.0773791*Temperature + 0.0149343*%Ethanol + 0.0289457*Time + 0.000646169*Temperature^2 + 0.000122917*Temperature*%Ethanol + 0.000787037*Temperature*Time + 0.000353879*%Ethanol^2 + 0.00284722*%Ethanol*Time + 0.000570456*Time^2

SAC = 3.00371 + 0.0220656*Temperature + 0.000142443*%Ethanol + 0.00908723*Time + 0.000147241*Temperature^2 + 0.0000425Temperature*%Ethanol + 0.000477778*Temperature*Time + 0.000151293*%Ethanol^2 + 0.00123056*%Ethanol*Time + 0.00340656*Time^2

Figure S1. Mathematical models for TPC (mg GAE/g DW) and SAC (mg/g DW).

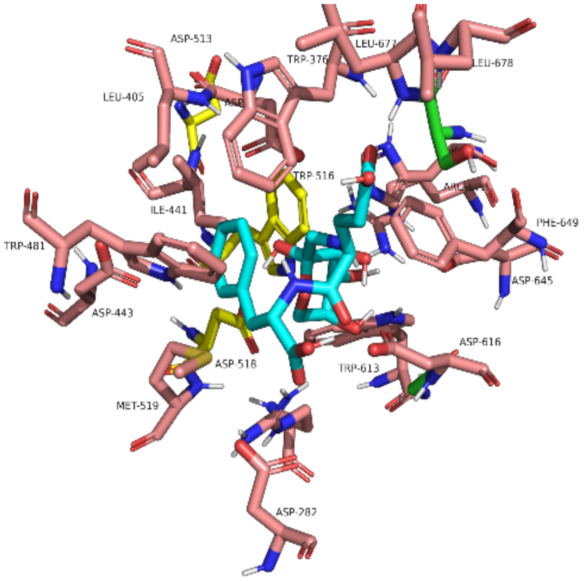

Figure S2. N-fructosyl-glutamylphenylalanine (cyan) forms hydrogen bonds between the N-fructosyl hydroxyl groups and the active site residue Asp-518 (yellow).

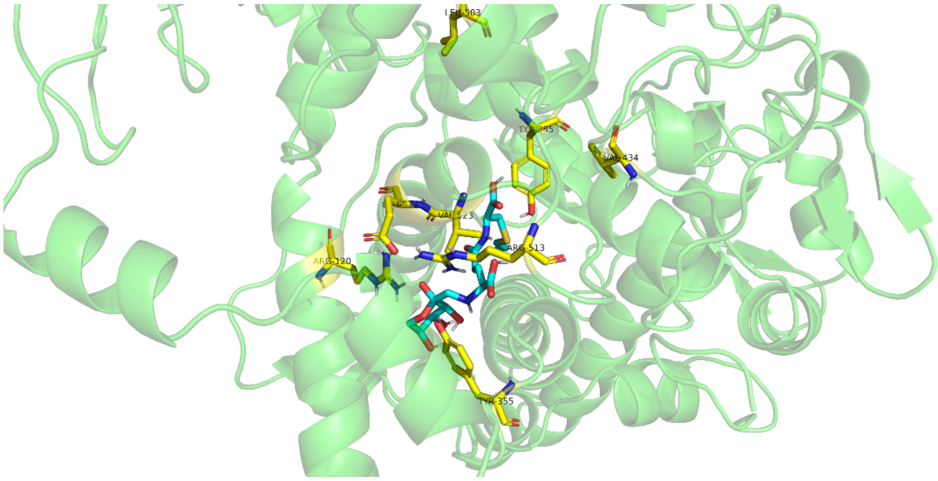

Figure S3. Interaction of N-fructosyl-glutamyl-S-(1-propenyl) cysteine (cyan) with active site residues Arg-120 and with Tyr-385 (yellow).

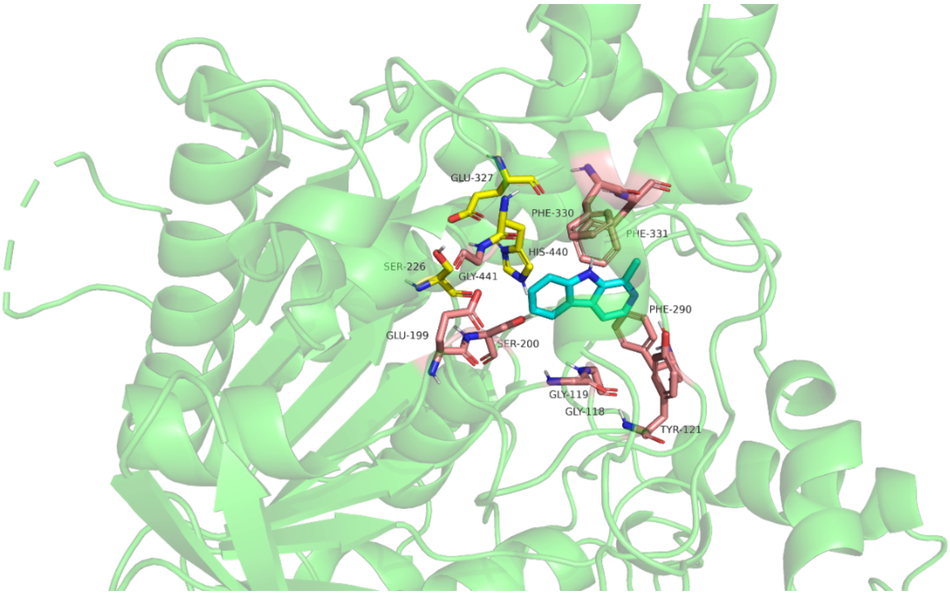

Figure S4. Interaction of Harmane molecule (cyan) with the His-440 residue (yellow). At this position, Harmane remains shielded by several aromatic residues.
